# Impulsivity and Risk-Taking Behavior in School-Going Adolescents

**DOI:** 10.7759/cureus.40728

**Published:** 2023-06-21

**Authors:** Ujjwal Soni, Rahul Sharma, Marisha Sharma, Ekta Khurana, Jayesh Chopra, Dhawani Julka, Nikhil Gaur

**Affiliations:** 1 Medicine, University College of Medical Sciences, New Delhi, IND; 2 Community Medicine, University College of Medical Sciences, New Delhi, IND; 3 Paediatrics, Lady Hardinge Medical College, New Delhi, IND; 4 Clinical Psychology, Gautam Buddha University, Greater Noida, IND

**Keywords:** pediatric psychology, adolescent and sexual health, impulsive behavior, child and adolescent psychiatry, risk-taking

## Abstract

Introduction

Impulsivity (or impulsiveness) and risk-taking behavior are significant concerns as the adolescent population is at a higher risk of injuries and violence, unhealthy sexual behaviors, and drug- and alcohol-related problems. The early identification of these traits in adolescents can prove beneficial through timely interventions. This study was conducted to assess impulsive behavior and risk-taking behavior among school-going adolescents in New Delhi, India, and to study the association, if any, between the two.

Methodology

A cross-sectional study was conducted among 571 students of classes 9th-10th in three randomly selected schools in a part of Delhi, India. Barratt Impulsiveness Scale - Brief (BIS-Brief) was used to evaluate impulsivity, and risk-taking behavior was assessed using the RT-18 tool.

Results

The majority (72.3%) of the 571 students were aged 14-15 years. Among the students, 56.0% were males. The impulsivity score obtained ranged from 8 to 30, with a mean score of 15.7 (SD ±4.1). The risk-taking score ranged from 2 to 18, with a mean score of 9.9 (SD ±2.9). Impulsivity was seen to be significantly higher among the female students (p=0.004). The risk-taking behavior was significantly higher among the students from government schools, among the females, and among those who used the internet more. There was a significant direct association between impulsivity and risk-taking behavior among the students (correlation coefficient 0.301, p<0.001).

Conclusion

The study results showed that the mean impulsivity and risk-taking scores were comparable to other studies in adolescent age groups done internationally using the same tools. Impulsivity and risk-taking behavior were both found to be higher among females. There was a significant direct association between impulsivity and risk-taking.

## Introduction

Adolescence, the period between childhood and adulthood, is characterized by significant changes in physical, cognitive, and emotional domains [1]. Impulsivity (or impulsiveness) is broadly defined as a tendency for rapid, unplanned reactions to internal or external stimuli without regard to the negative consequences of these reactions for the individual or others [2]. Impulsivity has been a focus of great interest both in the personality and clinical psychology literature due to its relevance for occupational and educational outcomes and its association with a wide range of psychiatric disorders, including substance use [3]. Disorders characterized by impulsivity include disorders of impulse control (intermittent explosive disorder, pyromania, kleptomania, etc.), paraphilias, sexual impulsions, sexual addictions, and personality disorders (borderline, antisocial, histrionic, and narcissistic personality disorders). Impulsivity has a substantial impact on both individuals and the society [4].

Risk-taking behavior is also the highest during adolescence, subsequently declining from youth to adulthood due to structural and functional changes in the brain [5]. It is a significant concern as the adolescent population is at a higher risk of injuries and violence, unhealthy sexual behaviors, and drug- and alcohol-related problems [6]. High-impulsive individuals have previously been perceived to be more risk-taking than low-impulsive [7]. The early identification of these traits in adolescents can prove beneficial through timely interventions.

Very few studies have been conducted to assess impulsivity and its relationship with risk-taking behavior among adolescents especially in the urban Indian population. This study was conducted with the aim to assess impulsive behavior and risk-taking behavior among school-going adolescents in Delhi, and to study the association, if any, between impulsivity and risk-taking behavior among the adolescent students.

## Materials and methods

This cross-sectional study was conducted over a period of three months in randomly selected schools in a part of New Delhi, the capital city of India. Three schools from the East Delhi district in Delhi were chosen randomly from a combined list of schools in the district using a table of random numbers for the purpose; the chosen schools included two government-run schools and one private-run school. Students of 9th and 10th standard of the selected schools participated in the study. The principals of the randomly selected schools were contacted and were informed about the purpose of the study; they were apprised of the fact that anonymity of the individual respondents would be maintained in the study. Written informed consent was obtained from the parents of the students and verbal assent from the students, prior to data collection. Ethical approval was obtained from the Institutional Ethics Committee, University College of Medical Sciences, University of Delhi, New Delhi, prior to the conduct of the study.

All students in the selected classes, present on the day of data collection, were eligible to participate, allowing for anonymous and voluntary participation. After obtaining students' assent, they were given time to fill up the questionnaire, after which the questionnaires were collected. Time was also given to address queries, if any, of the students.

Students enrolled in classes 9th-10th in the selected schools who provided verbal assent to participate in the study after obtaining written consent from their parents, and those who could understand, read and write in English were eligible to participate. English was the medium of instruction for these students. Students absent on the day of data collection were excluded.

The Barratt Impulsiveness Scale - Brief (BIS-Brief) was used to evaluate impulsivity. BIS is a questionnaire designed to assess the personality/behavioral construct of impulsivity, and is a widely cited instrument for the same. The original Barratt Impulsiveness Scale Version 11 (BIS-11) is composed of 30 items describing common impulsive or non-impulsive (for reverse-scored items) behaviors and preferences. Items are scored according to a Likert scale. The BIS-Brief is a shorter version of the BIS-11, that includes 8 of the original BIS-11 items. It includes question items such as “I plan tasks carefully”, “I do things without thinking”, to which the response options are 1 = rarely/never, 2 = occasionally, 3 = often, and 4 = always. Four of the eight items (1, 4, 5 and 6) are reverse scored. The item scores are summed and the total score obtained, ranging from 8 to 32, represents a uni-dimensional measure of impulsivity [8]. It is more appropriate for youth samples because it is less burdensome and omits items about activities not usually encountered by children and adolescents. Similar indices of concurrent validity for the BIS-11 total score have been demonstrated with the BIS-Brief score [9].

Risk-taking behavior was assessed using the RT-18 tool. This tool is a brief questionnaire developed to assess risk-taking among young adults. The questionnaire is comprised of 18 items such as “Do you usually think carefully before doing anything”, “Do you mostly speak before thinking things out” with responses to be given as ‘yes’ or ‘no’. The responses are scored as 1 = yes and 0 = no, with four of the 18 items (2, 9, 10 and 11) being reverse scored. The total score possible ranges from 0 to 18, with a higher score denoting higher risk-taking. It has two factors labelled as “level of risk-taking behavior” and “risk assessment”. The higher the score, the more risk-taking and less consideration of its consequences (risk assessment) [10]. Permission was obtained from the authors for both the tools, prior to their use in the study.

For socioeconomic status (SES), the assessment was done by a proxy measure using an “asset score” due to the problem of the validity of assessing family income from the school students. Students were asked about the possession of six assets by their family: bicycle, refrigerator, washing machine, computer (1 point each), motorcycle (2 points) and motor car (4 points). The total score was summed to give an asset score ranging from 0 to 10, with a higher score representing higher SES.

The data collected using the study tools was entered into a computer-based spreadsheet. The statistical analysis comprised calculating mean scores and distribution of the scores of impulsivity and risk-taking. Statistical tests of significance were then applied as appropriate for testing the significance of associations: chi-square test (Fischer’s exact test, if required) for the differences in proportions and t-test for differences in means. The correlation between continuous variables was tested using the calculation of Spearman’s correlation coefficient. Significance was taken at the level for p-values less than 0.05.

## Results

The total number of respondents in the survey was 571 students. The age of the students ranged from 12 to 18 years (mean 14.5 ± 1.0 years). The age was recorded in completed whole years only. The majority (413; 72.3%) of the students were aged 14-15 years. Among the students, 320 (56.0%) were males. The respondents included 237 (41.5%) from class 9th and 58.5% from class 10th. Most of the students were from government schools (468; 82.0%), the remaining being from private schools. The total number of family members in the students' families ranged from 2 to 19 (mean 5.5 ± 1.89 years). The number of brothers ranged from 0 to 5 (mean 1.1; median, mode both 1) and sisters from 0 to 7 (mean 1.2; median, mode both 1).

The internet use time on an average day, as reported by the students, ranged from 0 to 630 minutes (10.5 hours), with a median time of 60 minutes (interquartile range, or IQR: 0, 120 minutes). Nearly 73.4% of class 9th and 10th students accessed the internet for at least some time during an average day. The time spent on an average day playing computer/mobile games, as reported by the students, was a median of 30 minutes (IQR: 0, 70 minutes), and 489 (67.1%) of the students played computer/mobile games on an average day.

Father’s education level was unknown to 125 (21.9%) students. Among those who responded, it ranged from illiterate (4, 0.9%) to postgraduates (14, 3.1%). The mean number of years of the father’s education was 10.7 ± 3.1 years. Father’s occupation details were also collected as part of the study, but the responses were too varied to allow for any meaningful exploratory analysis.

Among the students, impulsivity was measured using the BIS-Brief. Each of the eight items of the scale had responses scored from 1 to 4 (Table 1). The total range of scores possible was 8 to 32, with a higher score implying higher impulsivity. As can be seen, a wide range was seen in the impulsivity scores of the students. Among the students, the actual score obtained ranged from 8 to 30, with a mean score of 15.7 (SD ±4.1). The risk-taking behavior among the students was assessed using the RT-18 scale. The score range possible was 0-18. Among the participants, the actual score obtained ranged from 2 to 18, with a mean score of 9.9 (SD ±2.9).

**Table 1:** Summary of the impulsivity and risk-taking scores among the respondents (n = 571) BIS-Brief, Barratt Impulsiveness Scale - Brief

	Impulsivity	Risk-taking
Tool used	BIS-Brief	RT-18
Range of score possible	8-32	0-18
Minimum score among the students	8	2
Maximum score among the students	30	18
Mean (SD) score	15.7 (4.1)	9.9 (2.9)
Median score	15	10

There were significant differences by gender in the study participants (Table 2). Females in the study were significantly more likely to belong to government schools, have a younger age, have lesser internet use and gaming use, have lower SES, larger family sizes, and a higher average number of brothers.

**Table 2:** Differences by gender among the characteristics of the students (n = 571)

Variable	Males (n = 320)	Females (n = 251)	p-value
Type of school			
Government	77.8%	87.3%	0.004
Private	22.2%	12.7%	
Class			
Ninth	42.8%	39.8%	0.47
Tenth	57.2%	60.2%	
Internet use			
One hour or less daily	50.3%	66.9%	<0.001
More than one hour daily	49.7%	33.1%	
Computer/mobile gaming			
One hour or less daily	65.0%	82.9%	<0.001
More than one hour daily	35.0%	17.1%	
	Mean values		
Age	14.6	14.4	0.037
Socioeconomic status (asset score)	4.7	4.0	0.004
Number of family members	5.4	5.7	0.028
Number of brothers	1.0	1.2	0.001
Number of sisters	1.2	1.1	0.52

The association of impulsivity with various characteristics of the students was tested as part of the study. The results are depicted in Table 3. Impulsivity was seen to be significantly higher among the female students compared to the males (p=0.004). There was no statistically significant association with the age of student, class at school, or internet usage. Furthermore, there was no association with the type of school, gaming use, or SES. The association of risk-taking with various characteristics of the participants was tested (Table 3). The risk-taking behavior was significantly higher among the students from government schools (compared to private schools), among females (compared to male students), and among those who used the internet more than an hour a day (compared to those using less than one hour daily). Risk-taking tendency decreased with the increasing number of sisters a participant had, though the correlation was not statistically significant. Risk-taking had a weak but statistically significant direct association with socioeconomic status (assessed by a 10-point asset scale). On testing for the association of risk-taking with the individual assets that comprised the SES asset scale, a statistically significant direct association was observed only with having a motorcycle in the family household (p=0.04; detailed results not shown).

**Table 3:** Association of impulsivity and risk-taking with various characteristics of the students (n = 571)

Grouping variable	Number of students	Impulsivity score		Risk-taking score	
	(by grouping variable category)	Mean score (±SD)	p-value	Mean score (±SD)	p-value
Type of school					
Government	468	15.7 (±3.96)	0.58	10.1 (±2.81)	<0.001
Private	103	15.9 (±4.46)		8.6 (±2.82)	
Gender					
Male	320	15.3 (±3.82)	0.004	9.2 (±2.80)	<0.001
Female	251	16.3 (±4.28)		10.8 (±2.74)	
Class					
9th	237	15.4 (±3.95)	0.12	9.96 (±3.08)	0.53
10th	334	16.0 (±4.12)		9.8 (±2.72)	
Internet use					
One hour or less daily	329	15.5 (±4.18)	0.11	9.7 (±2.89)	0.04
More than one hour daily	242	16.1 (±3.86)		10.2 (±2.83)	
Computer/mobile gaming					
One hour or less daily	416	15.8 (±4.05)	0.63	9.9 (±2.81)	0.37
More than one hour daily	155	15.6 (±4.07)		9.7 (±3.03)	
Variable		Spearman’s correlation coefficient		Spearman’s correlation coefficient	
Age	571	0.075	0.07	0.014	0.74
Socioeconomic status	571	-0.041	0.33	0.091	0.03
Number of family members	571	0.02	0.63	0.005	0.91
Number of brothers	571	0.063	0.14	0.054	0.19
Number of sisters	571	0.003	0.95	-0.07	0.097
Association of impulsivity with risk-taking					
Impulsivity score	571	-	-	0.301	<0.001

There was a significant direct association between impulsivity and risk-taking behavior among the students. The correlation coefficient between the impulsivity scale and the risk-taking scale scores was 0.301, and the association was statistically significant (p<0.001). As the impulsivity score increased, the risk-taking score was also significantly likely to be higher.

## Discussion

The present study included 571 students belonging to classes 9th-10th studying in three randomly selected schools that had students belonging to varied socioeconomic status. Only the adolescents belonging to these classes were included as the questionnaires were to be self-administered and would have been difficult to understand for the lower classes. The possession of all household assets asked about (bicycle, motorcycle, refrigerator, washing machine, computer, and personal car) was significantly higher among those in private school (compared to those in a government school), portraying the difference in socioeconomic backgrounds. Government school students had a significantly higher proportion of females (46.8%) than the private school (31.1%) in the present study (p=0.004), a fact that may be important while interpreting the later results.

The mean impulsivity score was found to be 15.7 (±4.1) in the current study. This is similar to the other international studies that have used the same BIS-Brief scale. The mean score was lower than the mean score of 19.03 found in a study in the USA [11]. However, this is expected as the USA study was conducted among young adults aged 18-25 years. Our mean impulsivity score was also lower than that found in a study of 1667 adolescents aged 12-17 years in Australia [12]. The study found mean scores of 19.0 and 17.7 in two sub-sets of their sample. While direct implications cannot be made, the adolescents sampled were those who had responded to an online survey about e-sports betting. Also, a significant proportion of these responders to the online survey had participated in e-sports betting within the last month. The impulsivity among these adolescents can be expected to be high, as the authors themselves had found impulsivity to be a significant predictor of past-month e-sports cash betting.

The mean risk-taking score among the adolescent students in our study was found to be 9.9 (±2.9). This was quite similar to the mean score seen among the adolescent population that used the same RT-18 scale. A study of high school students in Milan, Italy, found a mean score of 9.99 [13], while a study of adolescents aged 16-17 years in the UK found the mean score to be 9.1 [14].

Previous studies have consistently found a lower RT-18 mean risk-taking score among adults. Our mean score was higher than the risk-taking score (7.66 ± 4.6) observed in a study among adults aged 18-66 years in Manchester, United Kingdom, using the same scale [15]. This was also higher than the mean score (7.54 ± 3.5) seen among the healthy controls in a study of adults aged 18 years and above in Iran [16] and the score of 7.4 ± 3.9 found in a study of Australian young adults [17]. The longitudinal ‘CannTeen’ study conducted in the United Kingdom used the same RT-18 scale in both healthy adults and adolescents. The mean score among adolescents (9.1 ± 4.1) was quite comparable to our finding, while the score among adults (7.6 ± 4.1) was similar to the findings in other international studies just discussed [14]. It can be surmised that risk-taking is higher among adolescents than adults. Interestingly, the same CannTeen study also found that risk-taking was significantly higher among cannabis users (compared to healthy controls) in both adolescents and adults.

The association of impulsivity with various characteristics of the students showed some interesting findings. Impulsivity was significantly higher among female students. Men have been known to engage in impulsive and risky behaviors more frequently than women [18]. However, the relationship between impulsivity and gender is quite nuanced. The results of a meta-analysis indicated a stronger sex difference in motivational rather than effortful or executive forms of behavior control [18]. The results supported evolutionary and biological theories of risk-taking predicated on sex differences in punishment sensitivity. However, there is mixed evidence on the topic.

Another earlier meta-analysis found no difference between genders in novelty seeking, though women scored higher in reward dependence and harm avoidance [19]. It has been observed that males are generally thought to be more impulsive; however, the evidence for sex differences in impulsivity using objective behavioral measures is mixed. This review found that in laboratory animals, impulsive action was typically greater in males than females, whereas impulsive choice was typically greater in females. However, in humans, women discount more steeply than men, but sex differences in measures of impulsive action depend on tasks and subjects [20]. Another study exploring the sex difference in impulsivity observed that there seem to be sex differences in impulsivity, but these differences are more pronounced in childhood, and they are later subject to maturational and hormonal changes during adolescence and adulthood. Brain imaging studies, in fact, indicated that during adolescence, contrary to the evolutionary perspective hypothesis, young adolescent male individuals may be less vulnerable than age-matched females to risk- and reward-related maladaptive behaviors [21]. Previous studies have shown that adolescent samples with more females showed a larger impulsivity-risky sex relationship, suggesting that impulsivity may be a more important risk factor for risky sex among adolescent females [22]. A study of high school Italian students, using the Barratt Impulsiveness Scale, found no significant gender difference in impulsivity scores. Problematic internet use (using the Internet Addiction Test) was related significantly to higher impulsivity scores, a finding similar to that in the current study [23].

The risk-taking behavior was found to be higher among females, among those from government schools, and among those using the internet for more than one hour daily. A study of 13- to 20-year-old secondary school students from the United Kingdom found that male and female participants did not significantly differ in sensitivity to reward and impulsivity, but males had significantly higher risk-taking behavior [24]. It has been observed that epidemiological data indicate that risk behaviors are among the leading causes of adolescent morbidity and mortality worldwide. Consistent with this, laboratory-based studies of age differences in risk behavior demonstrate a peak in adolescence, suggesting that adolescents demonstrate a heightened inclination to take risks [25].

In the present study, both impulsivity and risk-taking scores were found to be significantly higher among female students, compared to males. However, there were significant differences in the socio-demographic characteristics of the study samples. The females were more likely to belong to lower SES, have a larger family size, and have a higher average number of siblings. All of these factors could have independently affected or modulated the role of gender. An interesting association of risk-taking was observed with one of the socioeconomic characteristics. While risk-taking was higher among females overall, the availability of a motorcycle at home was associated with a significantly higher risk-taking score among males only. It did not make a significant difference among females.

A strength of this study is the use of valid and reliable scales to measure impulsivity and risk-taking. Another strength is the inclusion of a large, diverse sample of students from different socioeconomic backgrounds. However, one limitation is the use of self-report measures, which may be subject to bias. Additionally, the cross-sectional design of the study does not allow for causal inferences to be drawn. The SES assessment has been done using an arbitrary ‘asset score’ rather than validated SES measures due to the problem of valid reporting of family income by school students. Another limitation is the lack of reliable knowledge about the psychiatric history (presence of attention deficit-hyperactivity disorder, autism spectrum disorder, mood disorders, traumatic brain injury, etc.) or family history, as well as academic functioning of these students. Future research could address these limitations by using a larger and more diverse sample and employing a longitudinal design to examine the development of impulsivity and risk-taking over time.

## Conclusions

The present study is among the initial ones to cover impulsivity and risk-taking behavior among the school-going adolescents in India. A significant direct association was found between impulsivity and risk-taking among the adolescent students. The results highlight the need for impulsivity reduction interventions among school-going adolescents with such interventions targeting specific sub-groups who are at a higher risk of risk-taking behaviors.
